# Guidelines for chest drain insertion do not protect relevant anatomical structures

**DOI:** 10.1186/cc12231

**Published:** 2013-03-19

**Authors:** J Bowness, P Kilgour, S Whiten, I Parkin, J Mooney, P Driscoll

**Affiliations:** 1 St Andrews University, St Andrews, Fife, UK; 2 University of Salford, UK

## Introduction

We have reported the risk of chest drain insertion inferior to the diaphragm when using current international guidelines [1]. Another complication is damage to significant peripheral nerves, such as the long thoracic nerve causing winging of the scapula [2]. We assessed these risks using: the European Trauma Course method, a patient's handbreadth below their axilla just anterior to the midaxillary line; the British Thoracic Society safe triangle [3]; and the Advanced Trauma Life Support (ATLS) course guidance [4].

## Methods

We used the above guidelines to place markers (representing chest drains) in the thoracic wall of 16 cadavers bilaterally (32 sides), 1 cm anterior to the midaxillary line. Subsequent dissection identified the course and termination of the long thoracic nerve, the site of lateral cutaneous branches of intercostal nerves, and their relation to the markers.

## Results

The long thoracic nerve was found in the fifth intercostal space in 16 of 32 cases, always in or posterior to the midaxillary line. Contrary to the description in *Grays' Anatomy *(40th edition) it terminated before the inferior border of serratus anterior. Most commonly it was found to end by branching in the fourth (right) or fifth (left) intercostal space (range third to sixth). Lateral cutaneous branches of intercostal nerves were found in the fifth intercostal space in 25 of 32 cases. Contrary to the description in *Last's Anatomy *(12th edition) they always passed anterior to the midaxillary line (and marker).

## Conclusion

Placement 1 cm anterior to the midaxillary line minimises risk to the long thoracic nerve and lateral cutaneous branches of intercostal nerves. We therefore conclude that not all areas of the British Thoracic Society safe triangle are indeed safe, and anteroposterior placement should follow the European Trauma Course and ATLS guidelines: just anterior to the midaxillary line (for example, 1 cm).
